# Biological In Vitro Evaluation of PIL Graft Conjugates: Cytotoxicity Characteristics

**DOI:** 10.3390/ijms22147741

**Published:** 2021-07-20

**Authors:** Katarzyna Niesyto, Wiktoria Łyżniak, Magdalena Skonieczna, Dorota Neugebauer

**Affiliations:** 1 Department of Physical Chemistry and Technology of Polymers, Faculty of Chemistry, Silesian University of Technology, 44-100 Gliwice, Poland; katarzyna.niesyto@polsl.pl (K.N.); wiktlyz002@student.polsl.pl (W.Ł.); 2 Department of Systems Biology and Engineering, Silesian University of Technology, Akademicka 16, 44-100 Gliwice, Poland; 3 Biotechnology Centre, Silesian University of Technology, Krzywoustego 8, 44-100 Gliwice, Poland

**Keywords:** graft copolymers, PIL, ionic conjugates, cytotoxicity, antituberculosis drugs

## Abstract

In vitro cytotoxicity of polymer-carriers, which in the side chains contain the cholinum ionic liquid units with chloride (Cl) or pharmaceutical anions dedicated for antituberculosis therapy, i.e., *p*-aminosalicylate (PAS) and clavulanate (CLV), was investigated. The carriers and drug conjugates were examined, in the concentration range of 3.125–100 μg/mL, against human bronchial epithelial cells (BEAS-2B) and adenocarcinomic human alveolar basal epithelial cells (A549) as an experimental model cancer cell line possibly coexisting in tuberculosis. The cytotoxicity was evaluated by MTT test and confluency index, as well as by the cytometric analyses, including Annexin-V FITC apoptosis assay. The polymer systems showed supporting activity towards the normal cells and no tumor progress, especially at the highest concentration (100 μg/mL). The analysis of cell death did not show meaningful changes in the case of the BEAS-2B, whereas in the A549 cell line, the cytostatic activity was observed, especially for the drug-free carriers, causing death in up to 80% of cells. This can be regulated by the polymer structure, including the content of cationic units, side-chain length and density, as well as the type and content of pharmaceutical anions. The results of MTT tests, confluency, as well as cytometric analyses, distinguished the polymer systems with Cl/PAS/CLV containing 26% of grafting degree and 43% of ionic units or 46% of grafting degree and 18% of ionic units as the optimal systems.

## 1. Introduction

In medicine, nano-sized materials can be applied as drug vehicles [1,2], where polymer-carriers improve a drug’s effect on the body through controlled release [3,4]. They are beneficial for limiting the side effects of low molecular weight medicine, i.e., exceeding the permissible dose of the drug [3]. In drug delivery systems (DDS), the bioactive substances can be loaded/encapsulated via physical interactions or chemically attached by a polymer matrix. The latter, known as the polymer-drug conjugates [5,6,7], are characterized by their stability, depending on the type of bonding, which requires the presence of specific sites in the polymer chain to ensure drug conjugation.

Ionic strength seems to be advantageous for ionic drug attachment [8,9]. In these cases, the carriers contain ionic groups, which are usually provided by ionic liquids (IL) as suitable (co)monomers introduced into the polymer chain [10,11,12]. Many of them are known as biocompatible, especially those based on choline chloride with cationic trimethylammonium groups [13,14,15,16,17]. Moreover, many poly(ionic liquid)s (PILs) are non-toxic in nature and show biological activity, which is desirable in medicine [18,19]. The carriers varied with structure and topology based on ILs have been designed via amphiphilic linear polymers, i.e., from vinylimidazolium [20], imidazolium [21], pyridinium [22] or guanidinium–type IL [23]. Numerous reports have been devoted to the use of phosphorylcholine IL as a co-monomer to obtain linear block copolymers [24,25], whereas those with graft topology and containing ionic units, i.e., imidazolium [26] or choline-type IL [12,27] in the side chains, have been investigated with significantly lower attention. In the case of the ionic polymer structure, drugs can be carried in ionic form as counterions, i.e., nicotinic, salicylic, ampicillin, naproxen, ibuprofenate anions [28,29,30], as well as in nonionic form as loaded guests [31], or both forms as the systems for combined therapy [32].

Drugs released from carriers travel along the body, where both the polymer and the active substance may have a direct effect on normal and diseased cells. Therefore, the optimal pharmacokinetics in correlation with the selective cytotoxicity of DDS is crucial in pharmaceutic therapy, where drug activity is expected toward diseased cells and can be utilized against tumor cells. Basic research to understand how a drug or carrier will respond in the body is supported by cell line assays [33]. Using in vitro models, with normal epithelial BEAS-2B and cancer A549 cells existing in a human respiratory system, the biological activity of tested compounds could be described. For further applications, most of the inhaled agents against different human diseases, such as tuberculosis, in the preliminary studies were tested using a standard cytotoxicity assay. Not only active substances but also components of drug delivery systems should be carefully studied in the first step of potential application. Contamination of the physiological microbiome of the human respiratory system with malignant pathogens could result in diseases. The main goal of a novel drug should be focused on the intelligent selectivity, with neutral action against healthy cells, cytostatic action against cancer cells and antimicrobial activity against resistant pathogens. 

Ionic drug-carrier conjugates based on graft copolymers containing ionic liquid units in the side chains, i.e., (2-trimethylammonium)ethyl methacrylate and methyl methacrylate copolymer (P(TMAMA-*co*-MMA)), have been designed to attach to ionic drugs, such as *p*-aminosalicylate (PAS) and clavulanate (CLV), which are common drugs used in lung diseases treatment, especially tuberculosis [27]. The infected cells are easily exposed to other pathogens, i.e., responsible for progress of cancer cells. Therefore, the drug systems were tested on human bronchial epithelial (BEAS-2B) as normal cells to check their non-toxic activity and adenocarcinomic human alveolar basal epithelial cells (A549) cells to exclude their supportive effect on tumor cells. PIL carriers varying with the type of counter ions, that is, Cl, PAS or CLV, were selected for the evaluation of cytotoxicity, which may be adjusted by possible correlations with the polymer structure parameters (content of TMAMA units, grafting degree and length of side chains). This verification was supported by in vitro cytotoxicity tests, that is, the colorimetric tests applying 3-(4,5-dimethylthiazol-2-yl)- 2,5-diphenyltetrazolium bromide (MTT), as well as cell cycle and apoptosis assays with the use of flow cytometry.

## 2. Results

**Figure 1:**
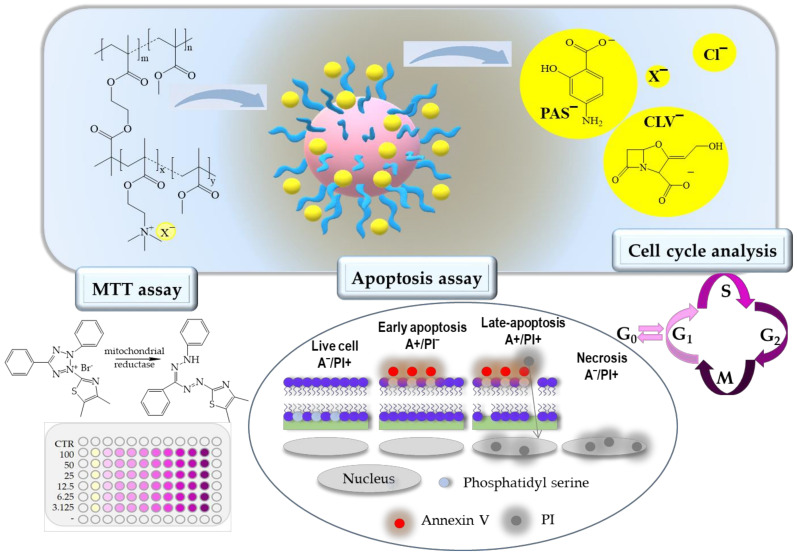
PIL and their conjugates with pharmaceutical anions for cytotoxicity evaluation.

Well-defined ionic graft copolymers, i.e., polymethacrylate backbones decorated by polymethacrylate grafts functionalized with ionic TMAMA moieties, were selected for biological studies, including both free carriers with chloride anions, as well as those carrying the pharmaceutical anions PAS and CLV as the cytostatic on cell lines and potentially antituberculosis drugs (Figure 1). Previous physicochemical reports have indicated that these systems are promising for drug delivery, in which the content of the anionic drug can be regulated by the content of TMAMA and then by the efficiency of anionic exchange of chloride to the drug [27]. Their self-assembling behavior in aqueous solutions has already been proven by the determination of critical micelle concentration (CMC), which reached values of 0.01–0.02 mg/mL for Cl based systems and slightly higher in the case of PAS/CLV containing systems (0.03–0.04 mg/mL). The evaluated carriers varied by backbone and side-chain length (n_mc_ and n_sc_, respectively), as well as number and content of ionic units (n_TMAMA_ and F_TMAMA_, respectively). Additionally, the conjugates differed with the type and content of ionic drugs (DC_PAS_, DC_CLV_). The structural characteristics of carriers I–IV, including polymer-drug conjugates, are presented in Table 1. Chosen systems were evaluated for their cytotoxicity towards human bronchial epithelial cells (BEAS-2B) to exclude toxic action on normal cells and adenocarcinomic human alveolar basal epithelial cells (A549), known as non-small lung carcinoma cell line, to exclude the expression of cancer activity. Some of the novel drugs could over-stimulate the cancerogenesis or cancer cells proliferation. The tests to exclude that activity were made.

### 2.1. MTT Cytotoxicity Assay

**Figure 2:**
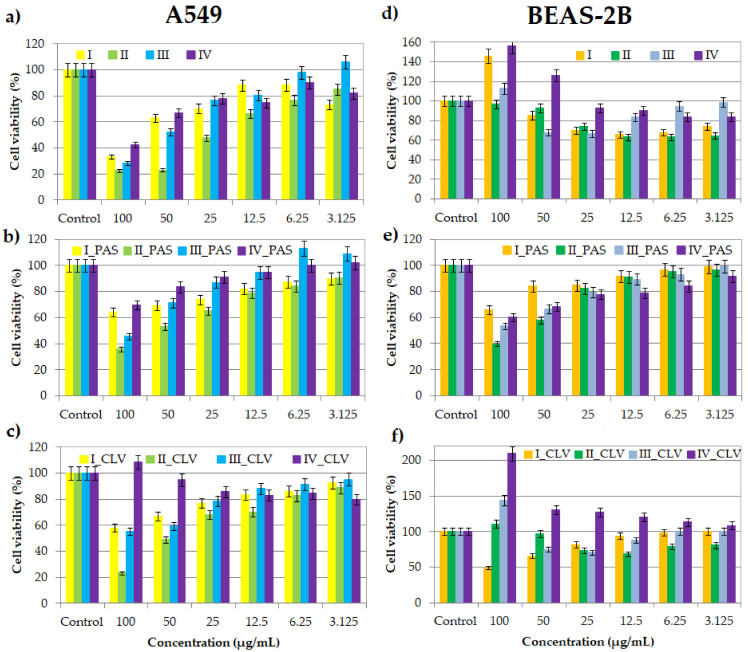
Cell viability of graft copolymers I, II, III and IV (**a**,**d**), and their conjugates with PAS (**b**,**e**) and CLV (**c**,**f**) at different concentrations for treatment of A549 and BEAS-2B cell lines, after 72 h of incubation in comparison to the untreated controls (100%).

One of the most popular cytotoxicity assays is the colorimetric MTT test, which allows the determination of the viability of cells after treatment and assesses a drug’s effect on proliferation. During the assay, due to the mitochondrial reductase, the reaction substrate—yellow water-soluble 3-(4,5-dimethylthiazol-2-yl)-2,5-diphenyltetrazolium bromide (MTT)—is modified by mammalian cells. The insoluble purple product (E,Z)-5-(4,5-dimethylthiazol-2-yl)- 1,3-diphenylformazan (formazan) directly appertains to the number of living cells [34,35,36,37]. Cell viability assays were performed at a series of concentrations (100–3.125 μg/mL) of nanocarriers I–IV, without pharmaceutical anions and their conjugates with PAS^−^ and CLV^−^. After treatment with all kinds of samples, poisoned cell lines were incubated for 72 h in standard conditions. Tested compounds showed cytotoxic activity against the tumor A549 cell line (Figure 2a–c). The cytotoxicity of carriers bearing Cl^−^ is higher in comparison to their conjugates with PAS^−^ or CLV^−^. Moreover, an increase in cytotoxicity measured by the percentage value of cell viability was observed with an increase in polymer concentration (100 vs. 3.125 μg/mL; I: 33% vs. 73%; II: 23% vs. 85%; III: 28 vs. 106%; IV: 42% vs. 82%). Similarly, for drug conjugates, cytotoxicity also increased with concentration. However, this effect was slightly lower than the case of the copolymers without the drug at a concentration of 100 μg/mL (PAS 64% (I), 36% (II), 46% (III), 70% (IV); CLV: 58%, 23%, 55%, 109%, respectively). In the case of the BEAS-2B cell line, treatment with nanocarriers bearing PAS^−^ caused the same dependence. At low concentrations, the negative effect was not observed, while higher drug concentrations interfere with proliferation (Figure 2e). After treatment with the copolymers and their CLV conjugates, a completely different relationship was observed (Figure 2d,f). The action of I_CLV, which induced an increase in toxicity with increasing concentration, was opposite to the IV_CLV system, which had a decreased toxic effect. In the range of low concentrations (3.125–12.5 μg/mL) of polymers I-III, and II_CLV and III_CLV conjugates, an increase in cytotoxicity was perceived with increasing concentration, whereas at higher concentrations (25–100 μg/mL), the toxic effect decreased. The highest tested concentrations of polymer systems did not cause cytotoxicity. These results indicated that as the concentration increases, the cells adapt, and their proliferation is improved. This phenomenon is called hormesis, which was described previously for doxorubicin-conjugates based on sugar core toward MCF-7 cell lines [38]. The inverse relationship of the action on BEAS-2B and A549 cell lines suggests that the compounds are selective for normal and cancer lung cells, which is a huge advantage.

**Figure 3:**
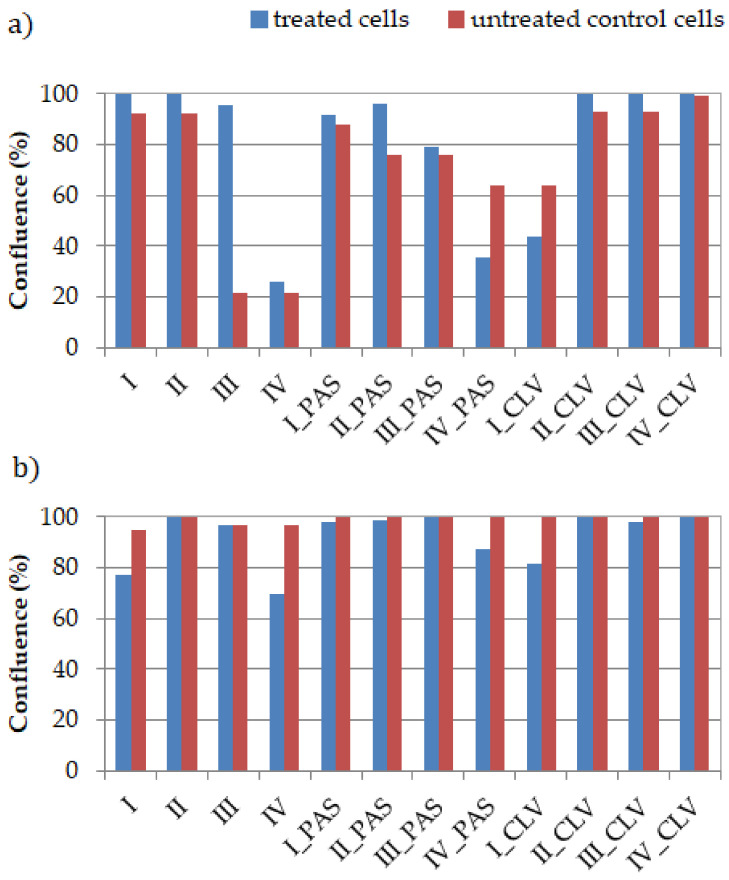
The confluence of polymer/conjugate (treated cells) in comparison to untreated control cells for (**a**) BEAS-2B and (**b**) A549 cell lines.

The percentage of the culture’s surface that is covered by cells, called confluence, was measured after 72 h of incubation at a concentration 100 μg/mL of the polymer sample. Generally, the confluence is regulated by programmed cell death (apoptosis). For the BEAS-2B cell line, in the case of copolymers bearing Cl^−^, an increase of the confluence of I, II, III and IV was observed in comparison to the control cells (Figure 3a). A similar relationship was observed for conjugates with PAS and CLV, where in most cases, the confluence reached almost 100%. In two cases, for I_CLV and IV_PAS, the confluence was lower than for the control cells, reaching ~40%. The increasing regularity means that adding these systems to the cells does not adversely affect the reproduction and activity of the BEAS-2B cells, and in most cases, this effect is even intensified. For the A549 cell line, the opposite relation was perceived (Figure 3b). The confluence for free carriers I, II, IV and conjugates I_CLV, III_CLV, I_PAS, II_PAS, III_PAS and IV_PAS decreased in comparison to their control cells. Adding of II, II_CLV and IV_CLV systems did not have any effect on proliferation.

**Figure 4:**
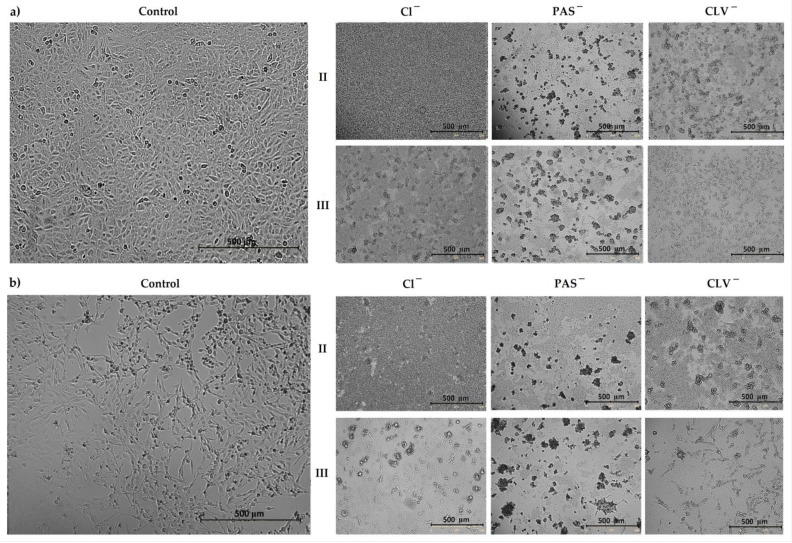
Microscopic images by Live Cell Analyzer for untreated control cells vs. treated (**a**) A549 and (**b**) BEAS-2B cells by polymer II and III with Cl^−^, PAS^−^ and CLV^−^ counterions. The scale bar represents 500 µm.

In relation to the MTT cytotoxicity assay, as well as the confluence of treated and untreated cells, including both normal (BEAS-2B) and tumor (A549) ones, the most optimal systems corresponded to II and III, with no exception to their PAS/CLV conjugates. They represented different structural parameters (Table 1), which means that there are more options to improve the cytotoxic activity in the designed polymer-carriers. In the case of sample II, lower grafting degree (26 mol.%) and densely distributed TMAMA units in the side chains reaching 43 mol.% are favorable for obtaining the suitable biological characteristics in relation to the tested cell lines. In turn, the lower TMAMA contribution (18 mol.%) in the copolymer III in combination with a larger number of side chains corresponding to a higher grafting degree (36%) also appeared advantageous in these studies. Both of them were capable of working selectively, showing cytotoxic activity for A549 and neutral for BEAS-2B. Microscopic images and a comparison of untreated and treated cells by polymer (I/II and III) and conjugates with PAS^−^ and CLV^−^ (I_PAS, I_CLV) are presented in Figure 4 and Figure S1.

### 2.2. Cytometric Analyses by Flow Cytometry

#### 2.2.1. Apoptosis Assay

Flow cytometry is currently used for observing changes in the cell cycle generated by drugs, apoptosis and cell cycle assay [39,40,41,42]. Cell death occurs as a result of the cytotoxic effect. Both the programmed death and the sudden, uncontrolled death may occur to cause cell damage (i.e., in response to the compound action). Flow cytometry allows understanding the processes in cells, permits the determination and analysis of the parameters of normal cells, as well as the cytotoxicity of compounds, especially due to tumor cells. The uncontrolled ability to reproduce, characteristic drug and apoptosis resistance are specific to cancerous cells. In this study, the Annexin-V apoptosis assay was performed to describe the type of cell death induced by a system solution with the drug using the respiratory BEAS-2B and A549 cell lines and 100 μg/mL of free carrier/conjugate dose.

**Figure 5:**
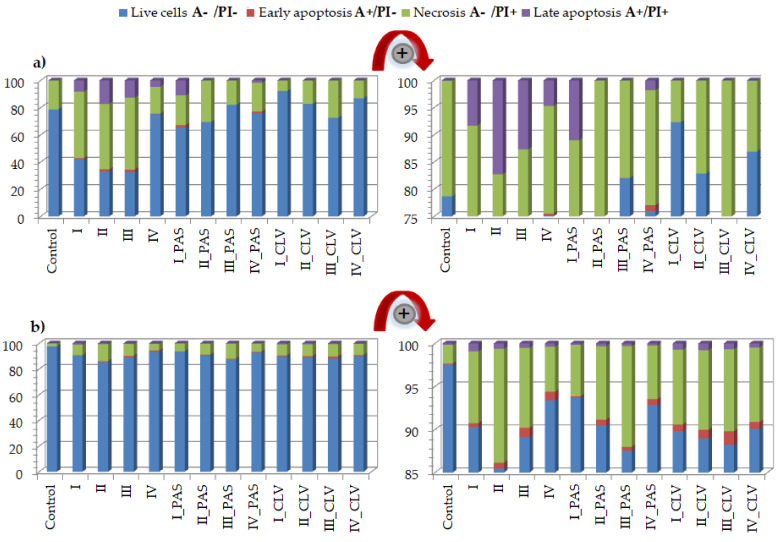
Annexin-V apoptosis assay results for (**a**) A549 and (**b**) BEAS-2B cell lines after the treatment of free carriers/conjugates and 72 h of incubation.

The A549 treatment effects indicated an increase in the necrotic state of cell death (Figure 5a, Table S1). The topology, number of side chains, and content of trimethylammonium groups, such as the type of conjugated anion, had a significant effect on cell death. The most visible changes were caused after treatment with free carriers I (A−/PI+ = 48.8%), II (A−/PI+ = 48.1%) and III (A−/PI+ = 53.0%) in comparison to control cells (A−/PI+ = 21.3%). Furthermore, an increase in the apoptotic state (A+/PI− and A+/PI+) was noticed for the treatment with free carriers (I: 9.2%; II: 19.0%; III: 14.6%; IV: 5.0%), where for the control cells, it was equal to 0.04%. Most cells survived after treatment with IV, similar to CTR cells (A−/PI− = 75.2%, 78.66%, respectively). The addition of PAS^−^ did not have a large impact on the change in the number of living cells. In the case of I_PAS and IV_PAS, the apoptosis phase (A+/PI− and A+/PI+) increased to 12.8% and 2.9%, respectively. Similarly, the treatment with CLV systems resulted in adaptation and increased cell survival. Generally, the free carriers containing trimethylammonium groups with chloride counterions showed anti-tumor action against the A549 cell line, with an exception for IV, which can be explained by the low content of TMAMA units in relatively short side chains densely grafted on the backbone. This effect was also reduced by the presence of pharmaceutical anions, such as PAS, and especially CLV.

**Figure 6:**
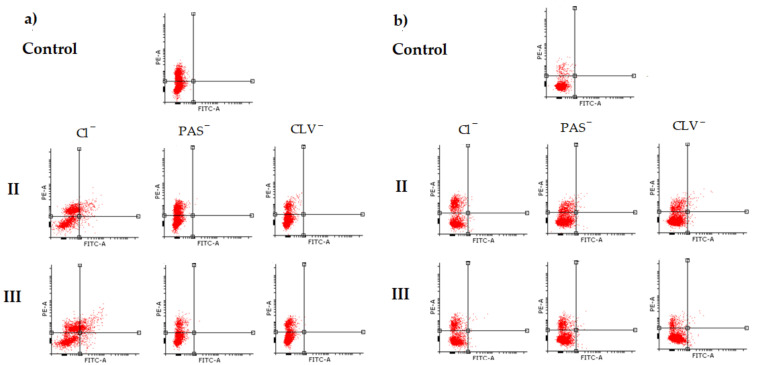
Representative plots of II and III cell populations determined by flow cytometric analysis in (**a**) A549 and (**b**) BEAS-2B cell lines.

The results for the BEAS-2B cell line also demonstrated the increase of necrotic state in comparison to the control (CTR A−/PI+ = 2.2%) (Figure 5b, Table S2). The changes were especially noticeable for cells treated by free carriers II, III and their conjugates II_PAS, III_PAS, II_CLV and III_CLV. A slightly lower effect was noticed for graft copolymers I and IV bearing Cl^−^, PAS^−^ and CLV^−^, where the necrosis was two to four times higher than in the control cells. However, the CLV anions had a greater effect on necrotic death. In each system, an increase in the apoptotic state was observed; nevertheless, the PAS conjugates had a minor effect (Figure 4b zoom in). Most importantly, these systems had no significant effect on the alteration of cell survival (A−/PI− = 85.5%−93.7%; whereas CTR A−/PI− = 97.5%), which suggests that tested carriers are non-toxic against BEAS-2B cell lines. The effects of polymeric carriers and their conjugates on cells are presented on representative plots of cell populations in Figure 6 and Figure S2.

#### 2.2.2. Cell Cycle Analysis

**Figure 7:**
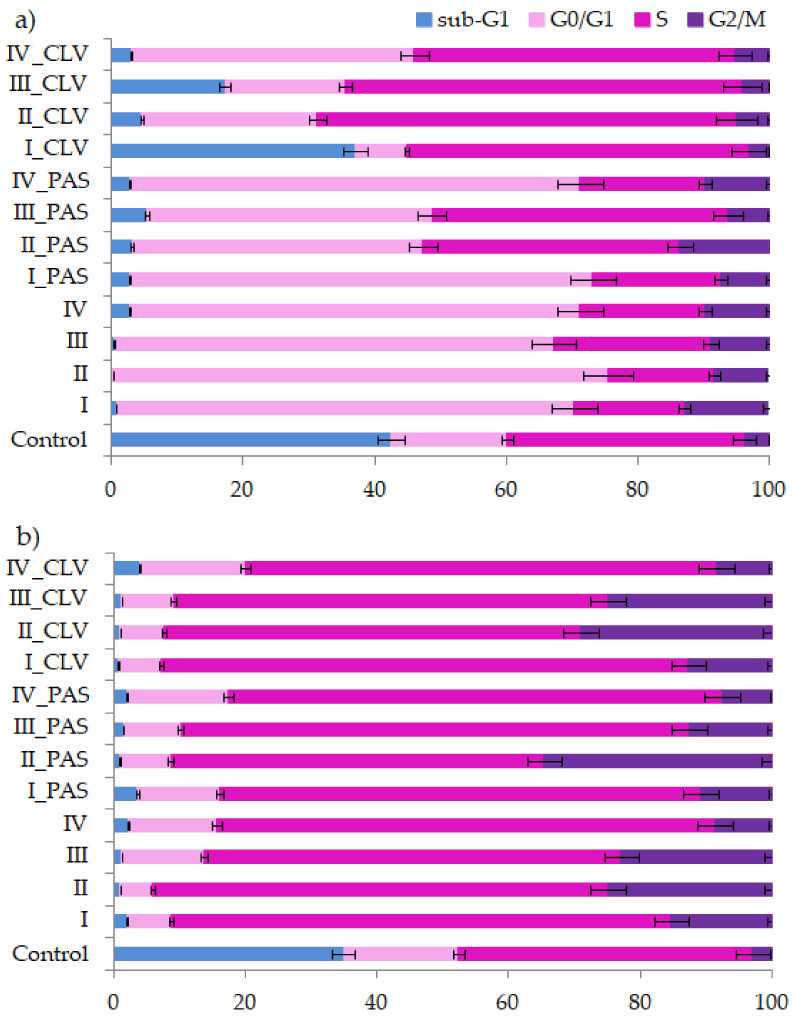
Cell cycle analysis of (**a**) A549 and (**b**) BEAS-2B cell lines treated by carriers I–IV or conjugates with PAS and CLV.

Cytometric methods allow for the determination of the polymer sample effect on the cell cycle, which goes through several phases (Figure 7). The phases start at zero, which is the resting phase (G0), then proceed to the cell growth phases defined as cellular division and beginning of DNA synthesis (G1), then to replication (S) and mitosis start (I), ending in mitosis of cells (M). Cell cycle analysis was performed in A549 and BEAS-2B lines treated by free carriers and their conjugates with PAS or CLV in one dosage (100 μg/mL) and incubated for 72 h. The cell lines were characterized by a natural rapid proliferation rate, and the extinction of cell cultures occurred in untreated control cell wells due to the crowding and contact inhibition of cells after 72 h. Therefore, for both A549 and BEAS-2B, many cells died and appeared in the sub-G1 phase.

In the case of the treated cells, the blockade of the cycle followed, and the G0/G1 and S phases increased relative to the untreated control. Those changes mainly proved that the compounds were not cytotoxic to the tested cells. Significant growth of the G0/G1 phase was also noted for the A549 cell line. The arrest in this phase means that these compounds act as cytostatics and cause cell cycle disorders. The smallest effect was supported by CLV conjugates, while the cells have been trapped in the S phase. Similarly, the BEAS-2B cells were arrested in the S phase after treatment by all systems. Because of the polymer system action, which could act as an intercalator, the replication was blocked. Nevertheless, in the S phase, there is a chance that the repair systems of cells and cell division will take place. The I/M phase did not change significantly after drug treatment for both A549 and BEAS-2B lines. The detailed data of cell cycles are presented in Tables S3 and S4.

## 3. Materials and Methods

### 3.1. Materials

Sodium phosphate buffer saline (PBS, pH = 7.4), DMEM-F12 medium, 3-(4,5-dimethylthiazol-2-yl)-2,5- diphenyltetrazolium bromide (MTT) and trypsin were received from Aldrich (Poznań, Poland). Propidium iodide solution (PI, BD Biosciences, San Jose, CA, USA), Annexin-V apoptosis assay (BioLegend, San Diego, CA, USA), physiological saline (PBS without Ca and Mg, PAN-Biotech Gmbh, Aidenbach, Germany), fetal bovine serum (FBS, EURx, Gdańsk, Poland) and Annexin-V binding buffer (BD Biosciences, San Jose, CA, USA) were used without prior preparation. Human bronchial epithelial cells (BEAS-2B) and adenocarcinomic human alveolar basal epithelial cells (A549) were purchased from ATCC (Cat# ATCC^®^CRL-9609; Manassas, VA, USA). Graft copolymers containing TMAMA units with chloride counterions were synthesized by controlled atom transfer radical polymerization, whereas their conjugates with *p*-aminosalicylic (PAS) or clavulanic (CLV) anions were obtained by chloride anion exchange, according to previously described procedures [27].

### 3.2. Characterization

Viability monitoring, cell density and confluence analysis were performed using Live Cell Analyzer (JuLI™ Br; NanoEnTek Inc., Seoul, Korea). The cytotoxicity was evaluated by measuring the absorbance of the formazan product at 570 nm with the use of a microplate reader (Epoch, BioTek, Winooski, VT, USA). Apoptosis and cell cycle analysis were carried out with the use of an Aria III flow cytometer (Becton Dickinson; Franklin Lakes, NJ, USA). Cytometric analyses were performed using PE configuration (547 nm excitation; emission: 585 nm) or FITC configuration (488 nm excitation; emission: LP mirror 503, BP filter 530/30).

### 3.3. Cell Culture

Cells were grown in a DMEM-F12 medium in sterile culture bottles (75 cm^2^ of culture area) with 10% (*v*/*v*) FBS at 37 °C in the incubator (humidified atmosphere with 5% CO_2_). The cell cultures were well placed in a 96-well plate in case of MTT tests (10,000 cells per well), and 6-well plate in case of cell cycle analysis and apoptosis assay (100,000 cells per well).

### 3.4. MTT Cytotoxicity Assay

A total of 10,000 cells were placed into 96-well plates in 0.2 mL of medium 24 h before adding polymer systems. Controls were prepared in the first row and outer columns of wells. A series of dilutions of the inoculated compounds were prepared in the remaining wells (3125–100 μg/mL). The treated and untreated (control) cells were incubated for 72 h in standard conditions. Then, the solutions were removed, and 50 μL of MTT solution (0.5 mg/mL in RPMI 1640 without phenol red) were placed into each well. After incubation (1–2 h), the MTT solution was taken out of the wells. Created formazan crystals were dissolved in 75 μL of isopropanol/HCl mixture (*v*/*v* 1/0.04). The cytotoxicity was evaluated by measuring the absorbance of the formazan product at 570 nm with the use of a microplate reader. Readings were repeated three times (six technical repetitions for each concentration). The results were presented as the percentage fraction of the control. The cell density estimation, viability monitoring and confluence analysis were carried out with the use of a Live Cell Analyzer. After 72 h of incubation, the microscopic images were taken of treated and untreated cells.

### 3.5. Cytometric Analyses by Flow Cytometry

A total of 100,000 cells were placed into 6-well plates in 2 mL of medium for 24 h before treatment. Then, 2 mL of the polymer/conjugate sample was placed into each well and incubated for 72 h.

Part of the solutions from the wells was placed into sterile vials and centrifuged for 3 min (0.6× *g*, RT). Then 50 μL of cold Annexin-V labeling buffer and 2.5 μL of FITC-labeled Annexin-V antibody were added to the pellet, and resuspended cells were incubated for 30 min in darkness. Next, the 250 μL of Annexin-V labeling buffer was added. An Annexin-V apoptosis assay was performed immediately using an Aria III flow cytometer.

For cell cycle analysis, the rest of the cell suspension collected from wells were centrifuged, and the supernatant was removed. Then, 250 μL of hypotonic buffer was added to the pellet (hypotonic buffer comprised PI 100 μg/mL in PBS; 5 mg/L of citric acid; 1:9 Triton-X solution; RNase 100 μg/mL in PBS from Sigma, Poznań, Poland) and the samples were incubated for 15 min in a cold and dark environment. The DNA levels were assessed by fluorescence measurements via BD FACS Aria^TM^ III sorter (Becton, Dickinson and Company, Franklin Lakes, NJ, USA) using PE configuration.

## 4. Conclusions

In vitro cytotoxicity evaluation of choline graft, polymer-carriers and their ionic PAS and CLV conjugates was based on MTT, apoptosis assay and cell cycle analysis. These tests indicated a strong correlation between biological action and carrier structure, including the type of attached pharmaceutical anions. Polymer systems with selective activity caused a negative effect on the tumor (A549) cell line, while they did not trigger significant changes in the normal (BEAS-2B) cell line. Moreover, the cytometric analyses proved the specific course of action. During studies on the type of cell death, it was found that in comparison to the control cells, a greater number of A549 cells died, mainly through necrosis. In turn, these compounds had no meaningful impact on BEAS-2B cells. Additional confirmation was achieved by cell cycle determination. Such findings suggest the potential usage of novel drugs for respiratory system diseases because of a wide application against cancer cells or pathogens (originated structures were reported as antimicrobial). For further findings, the specific test for antituberculosis therapy using standard assays should be performed [43].

The investigated ionic graft copolymers and their conjugates, previously tested for physicochemical evaluation, and evaluated cytotoxicity in this report, fulfilled the basic criteria for drug delivery systems. They are promising carriers of ionic drugs, especially those with a higher content of ionic units at lower graft density or lower content of ionic units at higher graft density, which can be used in the future for the treatment of lung diseases, such as tuberculosis, given the required specialized biomedical assessments.

## Figures and Tables

**Table 1:** Characteristics of amphiphilic graft copolymers and conjugates with PAS^−^ and CLV^−^^a^.

No.	n_mc_	DG (mol.%)	n_SC_/n_TMAMA_	F_TMAMA_ (mol.%)	M_n_ × 10^−3^ (g/mol)	Ð	DC (mol.%)	
							PAS	CLV
I	183	26	24/5	21	168.6	1.90	63.9	78.6
II			35/15	43	273.1	1.31	36.2	100.0
III	259	46	65/12	18	1090.5	1.11	37.0	66.3
IV			28/11	39	583.5	-	36.5	88.7

where: n_mc_ is the number of units in the main chain, DG is grafting degree, n_sc_ and n_TMAMA_ are numbers of repeating units in side chains and TMAMA units, respectively, F_TMAMA_ is the content of ionic hydrophilic units in side chains, M_n_ is average molecular weight, Ð is dispersity index, DC is drug content; ^a^ procedures, calculations and more detailed physicochemical data are reported in ref. [27].

## Data Availability

Not applicable.

## Supplementary Materials

The following are available online at https://www.mdpi.com/article/10.3390/ijms22147741/s1, Figure S1. Microscopic images by Live Cell Analyzer of untreated control cells vs. treated A549 and BEAS-2B cells by polymer I and IV with Cl^−^, PAS^−^ and CLV^−^ counterions; Tables S1 and S2. Results of Annexin V apoptosis assay in A549 and BEAS-2B cells for control probe, PIL carriers varying with content of TMAMA units (25% and 50%) and grafting degree (26% and 46%), and their conjugates with PAS and CLV; Figure S2. Plots of I and IV cell populations determined by flow cytometric analysis in A549 and BEAS-2B cell line; Tables S3 and S4. Results of cell cycle analysis for control probe, PIL carriers varying with TMAMA content (25% and 50%) and grafting degree (26% and 46%) and their conjugates with PAS and CLV, in treatment of A549 and BEAS-2B cells.
