# Supplementary material for: Biological In Vitro Evaluation of PIL Graft Conjugates: Cytotoxicity Characteristics
Source: Int J Mol Sci. 2021 Jul 20;22(14):7741. doi: 10.3390/ijms22147741 (PMC8306113; doi:10.3390/ijms22147741)
Supplement: Supplementary file 1 [file ijms-22-07741-s001.zip › ijms-1313664-supplementary.pdf]

Biological In Vitro Evaluation of PIL Graft Conjugates: Cytotoxicity Characteristics

Katarzyna Niesyto [1], Wiktoria Łyżniak[1], Magdalena Skonieczna [2,3,]* and Dorota Neugebauer[1,]*

[1] Department of Physical Chemistry and Technology of Polymers, Faculty of Chemistry,
Silesian University of Technology, 44-100 Gliwice, Poland; katarzyna.niesyto@polsl.pl
[2] Department of Systems Biology and Engineering, Silesian University of Technology, Akademicka 16,
44-100 Gliwice, Poland
[3] Biotechnology Centre, Silesian University of Technology, Krzywoustego 8, 44-100 Gliwice, Poland
* Correspondence: Magdalena.Skonieczna@polsl.pl (M.S.); Dorota.Neugebauer@polsl.pl (D.N.)

Content:

Figure S1. Microscopic images by Live Cell Analyzer of untreated control cells vs treated (a) A549 and (b) BEAS-2B cells by polymer I and IV with Cl^{-}, PAS^{-} and CLV^{-} counterions;

Table S1. Results of Annexin V apoptosis assay in A549 cells for control probe, PIL carriers varying with content of TMAMA units (25% and 50%) and grafting degree (26% and 46%), and their conjugates with PAS and CLV;

Table S2. Results of Annexin V apoptosis assay in BEAS-2B cells for control probe, PIL carriers varying with content of TMAMA units (25% and 50%) and grafting degree (26% and 46%), and their conjugates with PAS and CLV;

Figure S2. Plots of I and IV cell populations determined by flow cytometric analysis in (a) A549 and (b) BEAS-2B cell line;

Table S3. Results of cell cycle analysis in A549 cells for control probe, PIL carriers varying with content of TMAMA units (25% and 50%) and grafting degree (26% and 46%), and their conjugates with PAS and CLV;

Table S4. Results of cell cycle analysis in BEAS-2B cells for control probe, PIL carriers varying with content of TMAMA units (25% and 50%) and grafting degree (26% and 46%), and their conjugates with PAS and CLV.

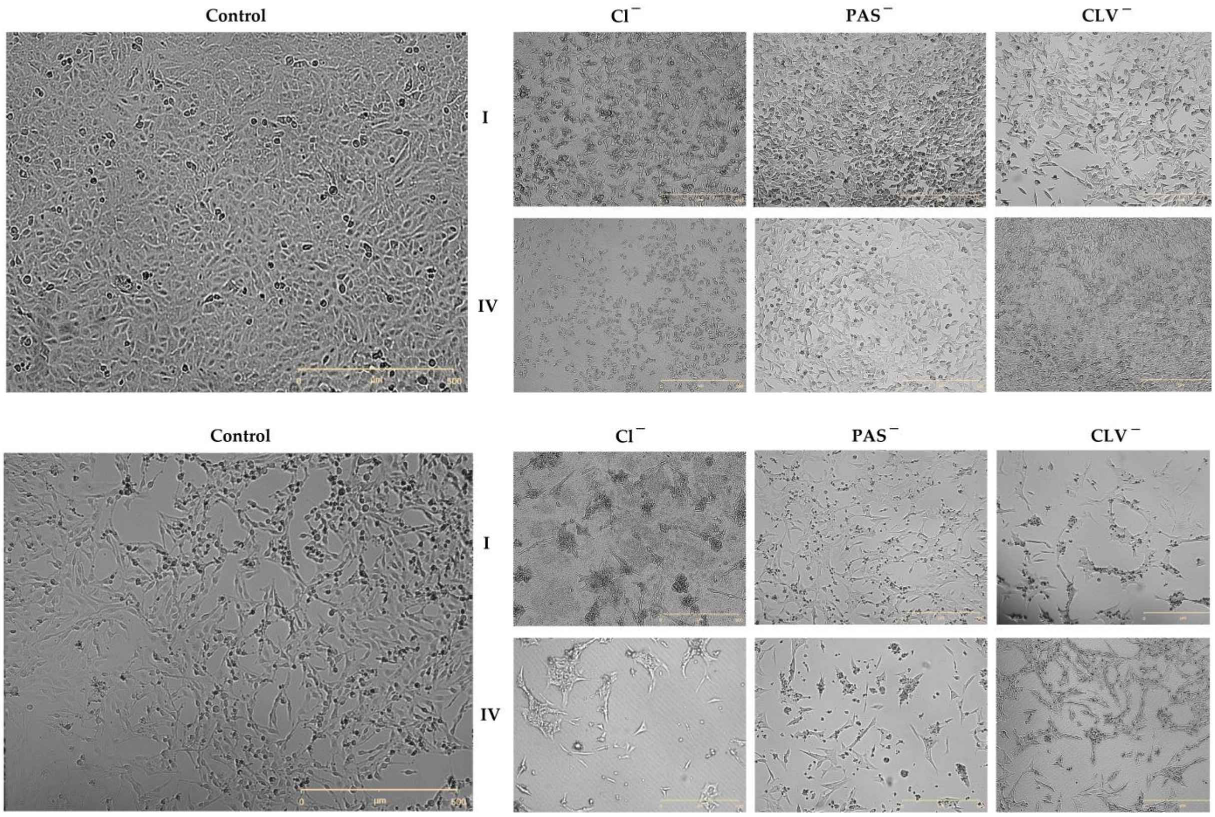

Figure S1. Microscopic images by Live Cell Analyzer of untreated control cells vs treated (a) A549 and (b) BEAS-2B cells by polymer I and IV with Cl^{-}, PAS^{-} and CLV^{-} counterions

Table S1. Results of Annexin V apoptosis assay in A549 cells for control probe, PIL carriers varying with content of TMAMA units (25% and 50%) and grafting degree (26% and 46%), and their conjugates with PAS and CLV

	Carrier				Conjugates with:							
					PAS				CLV			
	A− /PI− (%)	A+ /PI− (%)	A− /PI+ (%)	A+ /PI+ (%)	A− /PI− (%)	A+ /PI− (%)	A− /PI+ (%)	A+ /PI+ (%)	A− /PI− (%)	A+ /PI− (%)	A− /PI+ (%)	A+ /PI+ (%)
Control	78.7	0	21.3	0.04	78.7	0	21.3	0.04	78.7	0	21.3	0.04
I	42.2	0.8	48.8	8.3	65.3	1.8	21.9	11.0	92.3	0	7.6	0.03
II	32.9	1.7	48.1	17.3	69.5	0	30.5	0.03	82.9	0	17.1	0.03
III	32.4	1.9	53.0	12.7	82.0	0	17.9	0.01	72.6	0	27.4	0.01
IV	75.2	0.3	19.8	4.7	76.0	1	21.2	1.8	86.9	0	13.0	0.05

Table S2. Results of Annexin V apoptosis assay in BEAS-2B cells for control probe, PIL carriers varying with TMAMA content (25% and 50%) and grafting degree (26% and 46%), and their conjugates with PAS and CLV

	Carrier				Conjugates with:							
					PAS				CLV			
	A− /PI− (%)	A+ /PI− (%)	A− /PI+ (%)	A+ /PI+ (%)	A− /PI− (%)	A+ /PI− (%)	A− /PI+ (%)	A+ /PI+ (%)	A− /PI− (%)	A+ /PI− (%)	A− /PI+ (%)	A+ /PI+ (%)
Control	97.5	0.1	2.2	0.2	97.5	0.1	2.2	0.2	97.5	0.1	2.2	2.2
I	90.3	0.5	8.3	0.9	93.7	0.1	6.0	0.1	89.8	0.8	8.7	0.7
II	85.5	0.7	13.2	0.6	90.5	0.7	9.6	0.3	89.0	1.0	9.3	0.8
III	89.1	1.1	9.3	0.5	87.5	0.5	11.7	0.3	88.2	1.6	9.5	0.7
IV	93.4	1.0	5.2	0.4	92.8	0.7	6.2	0.3	90.1	0.8	8.7	0.5

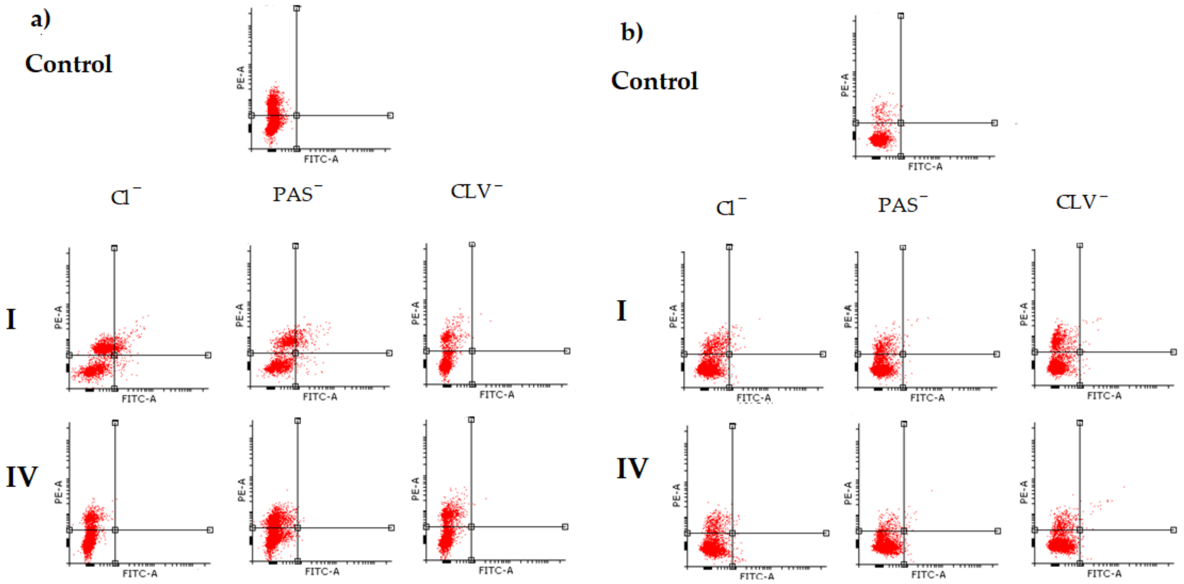

Figure S2. Plots of I and IV cell populations determined by flow cytometric analysis in (a) A549 and (b) BEAS-2B cell line.

Table S3. Results of cell cycle analysis in A549 cells for control probe, PIL carriers varying with content of TMAMA units (25% and 50%) and grafting degree (26% and 46%), and their conjugates with PAS and CLV.

	Carrier				Conjugates with:							
					PAS				CLV			
	Sub-G1 (%)	G0/G1 (%)	S (%)	G2/M (%)	Sub-G1 (%)	G0/G1 (%)	S (%)	G2/M (%)	Sub-G1 (%)	G0/G1 (%)	S (%)	G2/M (%)
Control	42.51	17.62	36.07	3.80	42.51	17.62	36.07	3.80	42.51	17.62	36.07	3.80
I	0.75	69.56	16.78	12.61	65.3	70.36	19.40	7.35	37.06	7.77	52.06	3.13
II	0.25	75.26	16.12	8.24	69.5	44.25	39.04	14.32	4.54	26.76	63.63	5.07
III	0.41	66.76	23.86	8.96	82.0	43.35	44.89	6.34	17.23	18.35	60.29	4.13
IV	2.82	68.40	18.95	9.82	76.0	68.40	18.95	9.82	2.95	43.04	48.75	5.24

Table S4. Results of cell cycle analysis in BEAS-2B cells for control probe, PIL carriers varying with content of TMAMA units (25% and 50%) and grafting degree (26% and 46%), and their conjugates with PAS and CLV.

	Carrier				Conjugates with:							
					PAS				CLV			
	Sub-G1 (%)	G0/G1 (%)	S (%)	G2/M (%)	Sub-G1 (%)	G0/G1 (%)	S (%)	G2/M (%)	Sub-G1 (%)	G0/G1 (%)	S (%)	G2/M (%)
Control	34.86	17.51	44.74	2.88	34.86	17.51	44.74	2.88	34.86	17.51	44.74	2.88
I	1.95	6.80	75.98	15.27	3.59	12.51	73.14	10.77	0.66	6.58	80.12	12.65
II	0.96	4.91	69.20	24.92	0.94	7.68	56.81	34.77	0.96	6.67	63.30	29.07
III	1.18	12.51	63.47	22.93	1.44	8.76	77.29	12.51	1.22	7.87	65.98	24.93
IV	2.12	13.52	75.78	8.57	1.92	15.48	75.13	7.47	3.92	16.06	71.56	8.46
